# Energy Consumption Forecasting for Smart Meters Using Extreme Learning Machine Ensemble

**DOI:** 10.3390/s21238096

**Published:** 2021-12-03

**Authors:** Paulo S. G. de Mattos Neto, João F. L. de Oliveira, Priscilla Bassetto, Hugo Valadares Siqueira, Luciano Barbosa, Emilly Pereira Alves, Manoel H. N. Marinho, Guilherme Ferretti Rissi, Fu Li

**Affiliations:** 1 Centro de Informática, Universidade Federal de Pernambuco, Recife 50740-560, Brazil; luciano@cin.ufpe.br; 2 Escola Politécnica de Pernambuco, Universidade de Pernambuco, Recife 50720-001, Brazil; fausto.lorenzato@upe.br (J.F.L.d.O.); epa@poli.br (E.P.A.); marinho75@poli.br (M.H.N.M.); 3 Graduate Program in Industrial Engineering, Federal University of Technology—Paraná, Ponta Grossa 84017-220, Brazil; pri_bass@hotmail.com (P.B.); hugosiqueira@utfpr.edu.br (H.V.S.); 4 Advanced Institute of Technology and Innovation (IATI), Recife 50751-310, Brazil; 5 CPFL Energia, Campinas, São Paulo 13087-397, Brazil; grissi@cpfl.com.br (G.F.R.); lifu@cpfl.com.br (F.L.)

**Keywords:** energy consumption, smart metering, forecasting, Box and Jenkins models, neural networks, ensembles

## Abstract

The employment of smart meters for energy consumption monitoring is essential for planning and management of power generation systems. In this context, forecasting energy consumption is a valuable asset for decision making, since it can improve the predictability of forthcoming demand to energy providers. In this work, we propose a data-driven ensemble that combines five single well-known models in the forecasting literature: a statistical linear autoregressive model and four artificial neural networks: (radial basis function, multilayer perceptron, extreme learning machines, and echo state networks). The proposed ensemble employs extreme learning machines as the combination model due to its simplicity, learning speed, and greater ability of generalization in comparison to other artificial neural networks. The experiments were conducted on real consumption data collected from a smart meter in a one-step-ahead forecasting scenario. The results using five different performance metrics demonstrate that our solution outperforms other statistical, machine learning, and ensembles models proposed in the literature.

## 1. Introduction

The interest in energy consumption in residential buildings has increased over the past years due to advances in home technology, economic technologies, and population growth [1]. Consumption profiles contribute with the elevated consumption since time indoors has increased due to the possibility of a home office [1]. Moreover, residential energy constitutes over 27% of global energy consumption [2,3] and over 40% of the consumption in United States and European Union [4].

Considering the amount of energy required in residential buildings, the employment of smart meters has become an important feature for planning and management of power generation systems [5]. Smart meters not only enable occupants to have insights of their own consumption patterns, but also provide useful information to energy suppliers in order to perform better planning of energy load. In this scenario, energy forecasting is considered an important tool for planning and decision making processes [6]. Its main challenge, however, is the high volatility of data concerning individual households. Consumption data can present different patterns since it can be influenced by external factors such as consumer profiles, weather, and the season of the year [7]. Moreover, the choice of an appropriate model can also affect the quality of forecasts. In fact, according to [2], only a 1% reduction in forecasting errors can have positive impacts in the economy.

Traditional linear forecasting models such as the autoregressive integrated moving average (ARIMA) and exponential smoothing (ETS) have being explored in the context of energy forecasting in smart meters [8,9]. ARIMA models assume a linear correlation structure among past data. As a result, it presents reduced accuracy when dealing with nonlinear data. In contrast, nonlinear models such as artificial neural networks (ANNs) and support vector machines (SVMs) can deal with nonlinear patterns in time series, but it may not deal with all patterns equally well due to problems of model misspecification, overfitting, and underfitting [10].

In the light of the limitations of linear and nonlinear models when individually employed and the high volatility characteristics presented in smart meter data, hybrid systems have being proposed in order to overcome such limitations and produce more accurate forecasts [2,4]. In particular, ensemble models take into consideration a pool of forecasting models, where the forecasts are combined in order to improve the forecasting quality. However, in order to achieve an improvement in performance, the pool of forecasting models must be accurate, uncorrelated, and diverse [11]. The intuition is that the strengths of a model may compensate the weaknesses of another, mitigating the risk of selecting a single unsuitable model.

There are two important steps in building ensemble systems: model generation and forecast combination. An ensemble can be composed of multiple models of the same method (homogeneous ensemble) or by different methods (heterogeneous ensemble). However, different diversity generation approaches can be employed to improve the accuracy of the ensemble. Diversity generation methods, such as bagging, perform random sampling bootstraps of the original training data in order to train each model. They are often employed in homogeneous ensembles [12]. In heterogeneous ensembles, the diversity is achieved through the employment of different forecasting methods. The combination of forecasts can be trainable or non trainable. Nontrainable combination models comprehend several statistical operators such as mean, median, and mode. Combination through the median and mode is less sensible to the presence of outliers than by the mean [13].

Trainable combinations can explore the flexibility of nonlinear models such as ANNs and SVRs in such a way that a meta-data is created based on the predictions produced by the pool of forecasters. In this way, the combination based on nonlinear trainable methods is trained on training data, allowing the combinator to generalize to unseen data, performing combinations of future forecasts of the base models. This strategy maps nonlinear relations between forecasts, but also brings an overhead of parameters, which might increase the computational complexity of the overall hybrid system.

Taking into consideration the volatility in energy consumption data from smart meters, the limitations of the forecasting models proposed in the literature and the computational complexity required in the training phase of nonlinear trainable combinations, this work proposes a heterogeneous ensemble composed of a pool of models with different characteristics, combined using an extreme learning machine (ELM) [14] model. We use ELM in our solution since it presents less computational complexity and fewer configurable parameters than traditional machine learning methods such as ANNs and SVRs [14].

More specifically, the proposed method presents the following advantages:

- The diversity of the ensemble is introduced by the employment of different forecasting methods such as autoregressive (AR), multilayer perceptron (MLP), extreme learning machine (ELM), radial basis function (RBF), and echo state network (ESN).
- The combination step employs an ELM model in order to map nonlinear relations between forecasts and to perform more accurate combinations.
- The proposed method is versatile, since different forecasting methods can be used in the pool, and then combined by the ELM.

Our solution is employed in the context of smart metering data and compared with traditional models and different forecasting combination methods proposed in the literature. The experiments are conducted taking into consideration different consumption patterns present in the data. The results demonstrated that the proposed method achieved better results than single models and other ensembles with different combinations.

The remainder of this article is structured as follows: Section 2 presents an overview of the related works, Section 3 details the proposed ensemble method and its components. The experimental setup and results are discussed in Section 4 and the Conclusion is presented in Section 5.

## 2. Related Work

The development of systems based on ML models has been highlighted in the energy forecasting area [15]. In this area, electricity load and energy consumption forecasts have received great attention due to their relationship to demand, supply, and environmental issues [16,17]. In general, electricity load forecasting tasks have a major impact on the planning, operating, and monitoring power systems. The accuracy of the forecasts can impact operation costs since an overestimation can increase the number of generators employed and produce an unnecessary reserve of electricity. The underestimation of electricity load can put at risk the system’s reliability due to insufficient load required to attend the demanding market [18]. In the same way, electricity consumption forecasting models can improve energy efficiency and sustainability in diverse sectors such as in residential buildings [19,20,21] and in industry [22,23].

In order to achieve accurate electricity load forecasts several machine learning (ML) models have been employed in this task [24,25,26]. Models such as ANNs based on wavelets [24], long short-term memory (LSTM), random forests [25], and ensembles [26] have been investigated.

Likewise, energy consumption forecasting systems based on ML models have been used in the literature. Culaba et al. [19] employed a hybrid system based on clustering and forecasting using *K*-Means and SVR models, respectively. Deep learning models such as convolution neural networks (CNN) were employed by [20] for energy consumption forecasts in the context of new buildings with few historical data. Pinto et al. [21] used ensemble models to forecast energy consumption in office buildings. Walther and Weigold [22] performed a systematic review of the literature on energy consumption forecasting models in the industry.

Considering the literature of energy consumption forecasting on smart metering data, several ML methods have been investigated. In this context, Gajowniczek and Zabkowski [27] employed MLP and SVR models to forecast the consumption on individual smart meters. For that, their solution extracts features related to the meter’s consumption history (e.g., average, maximum and minimum load) and the temperature inside the house. They argued that they do not perform a traditional time series modeling due to the high volatility of their data.

Zhukov et al. [28] investigated the effects of concept drift in smart grid analysis. A random forecast algorithm for concept drift was employed, and an ensemble using the weighted majority vote rule was used to combine the outputs of individual learners. The proposed method was compared to other algorithms in the concept drift detection context, obtaining promising results.

Electricity pricing and load forecasting are important tasks in smart grid structures due to the improvements of efficiency in the management of electric systems [17,29,30]. In this scenario, Heydari et al. [29] proposed a hybrid system based on variational mode decomposition (VMD), gravitational search algorithm (GSA), and general regression neural networks (GRNN). The VMD performs the series’s decomposition into several intrinsic mode functions (IMFS), while the GSA performs a feature selection in the time series. Furthermore, considering the importance of electricity load forecasting in electric systems, this task can also be performed in individual households through the employment of smart metering technologies [31,32]. In this way, Li et al. [33] employed a convolutional long short-term memory-based neural network with selected autoregressive features to improve forecasting accuracy. Fekri et al. [32] used deep learning models based on online adaptive recurrent neural networks, considering that energy consumption patterns may change over time. In addition, several load forecasting applications have been addressed, such as peak alert systems [34], where a modified support vector regression is employed, using smart meter data and weather data as input.

Another work that deals with smart metering forecast [7], investigated the effects of factors such as seasonality and weather condition for electricity consumption prediction using different ML models: regression trees, MLP and SVR. Their findings show that: regression trees obtain the lowest root mean squared error (RMSE) values in almost all evaluated scenarios; adding weather data does not improve the results; and a historical window of one year to train the models is enough to achieve low-error forecasts.

Sajjad et al. [35] propose a deep-learning model for hourly energy consumption forecast of appliances and houses. The input data is processed using min-max normalization or z-score standardization, which is fed into a convolutional neural network (CNN) followed by a recurrent neural network (RNN), specifically a gated recurrent unit (GRU). Finally, a dense layer on top of the GRU outputs the prediction. They do not provide, however, any details about their strategy of selecting the hyper-parameters of the network.

Similarly, Wang et al. [36] employ an long short-term memory (LSTM) model that outputs quantile probabilistic forecasts. For training, the network minimizes the average quantile loss for all quantiles. The input of the network is composed of the historical consumption, the day of the week and hour of the day of the data point to be predicted. Similar to [35], the process of selection of nodes and layers of the network is not presented.

In addition, hybrid systems have gained attention due to their ability to increase the accuracy of the single ML models [16,37]. These systems are developed aiming to overcome the limitations of single ML models regarding misspecification, overfitting, and underfitting [10]. In this sense, Somu et al. [38] employed the *K*-means clustering-based convolutional neural networks and long short term memory (*K*CNN-LSTM) to forecast energy consumption using data from smart meters. In this work, the *K*-means is employed to identify tendency and seasonal patterns in the time series, while the CNN-LSTM is used in the forecasting process.

Chou and Truong [39] proposed a hybrid system composed of four steps: linear time series modeling, nonlinear residual modeling, combination, and optimization. The parameter selection process for the models employed in the first three steps is performed through a Jellyfish Search (JS) optimization algorithm [40]. Bouktif et al. [41] employed a genetic algorithm (GA) and particle swarm optimization (PSO) to search for hyperparameters of the LSTM in load forecasting tasks.

The proposed hybrid system differs from the hybrid systems proposed in the literature since it employs a GA to perform the optimization of the residual forecasting model and the combination model. Furthermore, the optimization also selects the most relevant lags to reduce model complexity and enhance forecasting accuracy.

## 3. Proposed Ensemble Method

**Figure 1:**
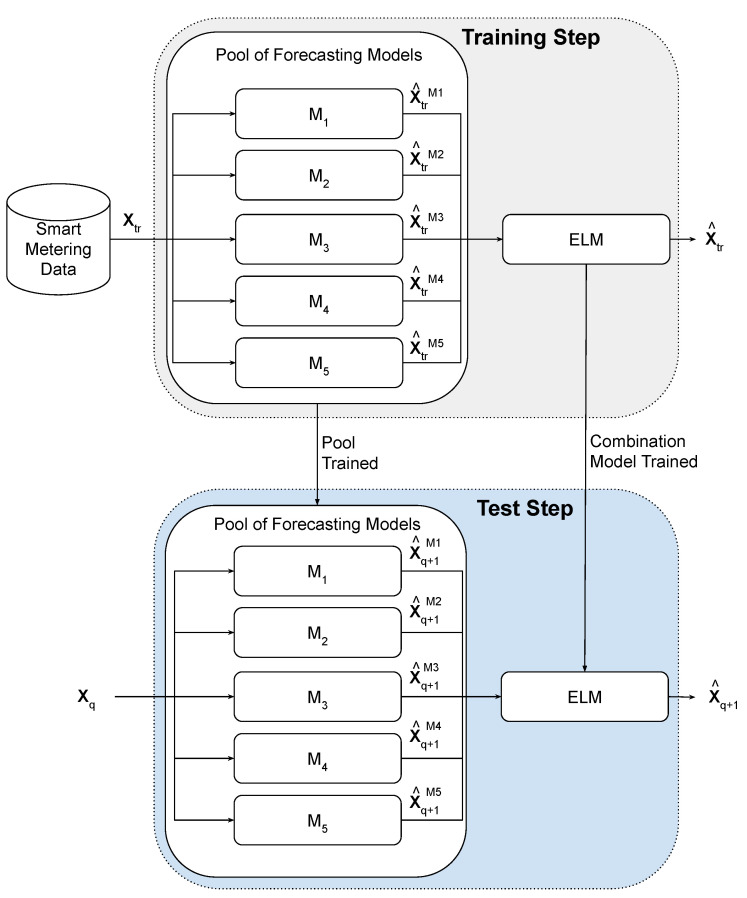
Model of the proposed ensemble.

Ensembles are elaborated in order to improve the final response from of the single trained models (specialists) combining their outputs [42]. The idea is that each single model presents a better performance for some subset of the input data. Hence, a combination model can use each best single output to generate a more accurate final response [43,44]. Figure 1 summarizes the general idea of the proposed ensemble, presenting its two main steps: training and test.

In the training step, the single and combination models are adjusted to improve some performance measure. First, the single models ($M_{1},M_{2},\ldots ,M_{5}$) are trained from training instances ($X_{tr}$) that contain the time lags of the time series and the respective desired output. Then, the combination models are trained to fuse the single models’ forecasts in order to minimize the difference between the desired output and the ensemble forecast according to the performance measure. Each combination model receives a data set that combines the forecasts of the training pattern of the single models for each desired output of $X_{tr}$.

In the test step, given an unseen test pattern $X_{q}$, every single model generates one forecast ${\hat{X}}_{q+1}^{M}$. This set of forecasts is then passed to the combination model, which generates the final forecast ${\hat{X}}_{q+1}$.

In our solution, the pool of single models employed by the proposed ensemble method comprises the statistical linear AR and four well-known ML models: MLP, ELM, RBF, and ESN [45] The AR model assumes a linear correlation structure in the data; therefore it can not perform nonlinear mappings. MLP, ELM, RBF, and ESN are flexible, data-driven, and able to perform nonlinear mappings. The MLP employs a multilayered architecture in order to learn from data, while the ELM uses a single hidden layer. The ESN has feedback loops of information in the hidden layer. The RBF is based on the locality learning principle since Gaussian functions are often employed as activation functions in the hidden units. Thus, these models represent different architectures in the energy consumption literature and were chosen due to promising results in time series forecasting tasks, especially those related to electricity [45,46,47,48,49].

In the proposal, the combination of the forecasts is performed using an ELM model. Therefore, since the combination is performed by a trainable method, the data set used for its training process is composed of the predictions of the pool ($X_{\mathrm{tr}}^{M1},X_{\mathrm{tr}}^{M2},X_{\mathrm{tr}}^{M3},X_{\mathrm{tr}}^{M4},X_{\mathrm{tr}}^{M5}$) and the target output value. After the training process is complete, the ELM model performs the combination of the forecasts achieved by the pool in the test set (${\hat{X}}_{q+1}^{M1},{\hat{X}}_{q+1}^{M2},$${\hat{X}}_{q+1}^{M3},{\hat{X}}_{q+1}^{M4},{\hat{X}}_{q+1}^{M5}$) to generate the final forecast of the system ${\hat{X}}_{q+1}$.

Considering that ML models may present problems such as model mispecification, overfitting, and underfitting [10], ensemble methods can further increase the accuracy of the base models through of combination approaches. The errors could be decomposed to assure an adequate performance of the method, it is a crucial condition that the single models also present accurate performance and diversity in the response [50,51,52].

Let $\bar{f}$ be the ensemble and $X_{q+1}$ be the target output, the overall forecasting error of the ensemble can be decomposed as presented in Equation (1), where the first term of the right hand side of the equation represents the difference between the forecast and the target output, which is often referred to as bias, whereas the latter term represents the stability of the model in unseen samples, and is often referred to as variance.

(1) $$E\left\{{\left(\bar{f}\left(X\right)-X_{q+1}\right)}^{2}\right\}={\left(E\left\{\bar{f}\left(X\right)\right\}-X_{q+1}\right)}^{2}+E\left\{{\left(\bar{f}\left(X\right)-E\left\{\bar{f}\left(X\right)\right\}\right)}^{2}\right\}.$$

The employment of ensemble methods through averaging forecasts allows a decrease of the variance without increasing the bias term, therefore increasing the generalization capacity of the forecasting system [53]. The literature presents many different combiners, such as average, weighted voting, and using ML methods [50,51,52].

The following sections describe in further details the single and combination models employed in the proposed ensemble method.

### 3.1. Single Model: Autoregressive Model

The autoregressive Model belongs to the framework of the linear Box and Jenkins methodology. It is one of the most widely known approaches due to its good results presented in the literature and simple parameters’ adjustment, with is based on a closed form solution [54].

Let $x_{t}$ be a sample of a time series, and $x_{t-p}$ its *p*-th lag (delay). We define the autoregressive model of order *p* (AR(p)) as the weighted combination of *p* lags of observation $x_{t}$, as in Equation (2) [54]:

Given any value $x_{t}$ of a time series, the delay *p* is defined with $x_{t-p}$. An autoregressive process of order *p* (AR(p)) is defined as the linear combination of *p* delays of observation $x_{t}$, with the addition of a white Gaussian noise $a_{t}$, as showed in Equation (2):

(2) $${\tilde{x}}_{t}=\phi_{1}{\tilde{x}}_{t-1}+\phi_{2}{\tilde{x}}_{t-2}+\ldots +\phi_{p}{\tilde{x}}_{t-p}+a_{t}$$

where $a_{t}$ are white Gaussian noises (shocks) or the inherent error of the prediction, ${\tilde{x}}_{t}=x_{t}-\mu$ (μ is the average of the series), $\phi_{p}$ is the weighting coefficient for the lag *p*.

The solution of the Yule–Walker equations is given in matrix form by Equation (3):

(3) $$\Phi_{p}=P_{p}^{-1}\rho_{p}$$

in which we can expand the elements as in Equation (4):

(4) $$P_{p}=\left[\begin{array}{cccc} 1 & \rho_{1} & \cdots & \rho_{p-1}\\ \rho_{1} & 1 & \cdots & \rho_{p}\\ \cdots & \cdots & \cdots & \cdots \\ \rho_{p-1} & \rho_{p-2} & \cdots & 1 \end{array}\right]\rho_{p}=\left[\begin{array}{c} \rho_{1}\\ \rho_{2}\\ \vdots \\ \rho_{p} \end{array}\right]\Phi_{p}=\left[\begin{array}{c} \phi_{1}\\ \phi_{2}\\ \vdots \\ \phi_{p} \end{array}\right]$$

where $\rho_{p}$ is the coefficients of the autocorrelation function of the series.

### 3.2. Single Model: Multilayer Perceptron (MLP)

Undoubtedly, the multilayer perceptron (MLP) is the most used artificial neural network architecture for nonlinear mapping due to its versatility and applicability [43]. Endowed of a set of artificial neurons organized in at least three multiple layers (input, hidden, and output layers), the MLP is a feedforward neural model.

The training of an MLP consists of tune the weights of the neuron to provide an adequate mapping between the inputs and the desired response [55,56]. The literature presents many methods to adjust an MLP, usually based on unconstrained nonlinear optimization. The most known method is the steepest decent algorithm in which the gradient vector is calculated using the backpropagation algorithm [57,58]. However, in this work we address the modified scaled conjugated gradient, a second order method [47].

The steps involved in the training of the MLP consists of two main iterative phases. The first one is a forward progression, in which the inputs data is propagated in the neural model until the achievement of the outputs. Next, the instant error regarding such outputs is calculated, using the desired output response. In the second step, the weights are adjusted from the output layer to the input layer, following the assumed optimization rule. In this sense, the error provided by the MLP in the next iteration is smaller. The kind of adjustment that uses a desired signal lies in the framework of the supervised training [59].

### 3.3. Single Model: Echo State Networks (ESN)

Designed in 2001, the echo state networks (ESN) are a kind of recurrent neural network since they have feedback loops of information in the hidden layer. This intrinsic characteristic may bring gains in the performance of the neural models when the inputs present temporal dependence, as in time series forecasting [60,61].

Recurrent models present different response depending on their internal state. The convergence proof of the ESN shows that the most recent inputs and the previous states influences more the output response of the network. Hence, Jaeger [60] used the term *echo* to describe the *echo state propriety* [62], which demonstrates the conditions for the network to present echo states.

Similar to MLP, the original ESN presents three layers. The hidden layer is named dynamic reservoir. This layer presents fully interconnected neurons, which are responsible to generate the nonlinear characteristic. The output layer combines the responses of the reservoir. Only the reservoir presents feedback loops in the original proposal. For each new input $u_{t+1}$ the internal states of the ESN are updated following Equation (5):

(5) $$x_{t+1}=f\left(W^{\mathrm{in}}u_{t+1}+Wx_{t}\right)$$

where $x_{t+1}$ are the states in time t+1, $f\left(\cdot \right)=(f_{1}\left(\cdot \right),f_{2}\left(\cdot \right),f_{3}\left(\cdot \right),\ldots ,f_{N}\left(\cdot \right))$ the activations of reservoir neurons, and $W^{\mathrm{in}}$ the weights of the input layer.

The output response $y_{t+1}$ is according to Equation (6):

(6) $$y_{t+1}=W^{\mathrm{out}}x_{t+1}$$

in which $W^{\mathrm{out}}\in R^{L\times N}$ is the matrix containing all weights of the output layer, and *L* is the number outputs.

The weights of an ESN in the dynamic reservoir are not adjusted in the training phase. The Moore–Penrose pseudo-inverse operator (Equation (9)) is used to determine the coefficients of $W^{\mathrm{out}}$.

To create the dynamic reservoir, we use the original idea from Jaeger [60]. In this case, the weight matrix is composed of 3 possible values, which are randomly chosen according to the following probabilities:

(7) $$W_{ki}^{in}=\left\{\begin{array}{c} 0.4\;\mathrm{with}a\mathrm{probability}\mathrm{of}0.025\\ -0.4\;\mathrm{with}a\mathrm{probability}\mathrm{of}0.025\\ 0\;\mathrm{with}a\mathrm{probability}\mathrm{of}0.95 \end{array}\right.$$

### 3.4. Single Model: Radial Basis Function Network (RBF)

The radial basis function networks (RBF) is another classic ANN architecture. It has only one hidden and one output layer. In the hidden layer, all kernel functions (activation) are radial-based, and the Gaussian function is the most used [59]. This function is in Equation (8):

(8) $$\varphi \left(u\right)=e^{-\frac{{\left(u-c\right)}^{2}}{2\sigma^{2}}}$$

where *c* the center of Gaussian, and $\sigma^{2}$ the corresponding variance which is a function of the center position.

To adjust an RBF, it is necessary to follow two steps. In the first stage the synaptic weights of the intermediate layer are determined, and the center is adjusted to the value of the base variance of each function. This stage is adjusted by an unsupervised clustering approach [59]. In this work, we used the K-Medoids method. The second step involves the calculation of the weights of the output layer [63]. There are some possible approaches to perform this task. The most usual is the use of the backpropagation algorithm, as in MLP. Another possibility is the direct application of the Moore–Penrose pseudo-inverse operator (Equation (9)) [59], which is the one we chose in this work.

### 3.5. Single Model: Extreme Learning Machine (ELM)

Extreme learning machine (ELM) is a feedforward neural network architecture similar to the traditional MLP. The main difference lies in the training procedure, while we adjust all neural weights in the MLP, in the ELM, just the output layer is trained using a supervised approach. In addition, the ELM presents just one hidden layer [14].

In this sense, the neurons in the hidden layer are randomly generated and stands untuned. The training of an ELM is summarized in finding the weights of the output layer $W^{\mathrm{out}}$ that lead the networks response to the smallest error regarding the desired output d [14]. The usual way to solve this task is use a closed form solution, the Moore–Penrose pseudo-inverse operator. Besides the small computational cost involved in its application, the operator ensures minimum mean square error between the network response and the desired output. This solution is in Equation (9):

(9) $$W^{\mathrm{out}}={\left(X_{\mathrm{hid}}^{T}X_{\mathrm{hid}}\right)}^{-1}X_{\mathrm{hid}}^{T}d$$

in which $X_{\mathrm{hid}}\in R^{|x|\times NN}$ is the matrix containing all outputs of the hidden layer for the training set, and NN is the number of neurons in the output layer [14].

## 4. Experimental Evaluation

In the next sections, the experimental protocol and results are described. Section 4.1 presents the data set used in the experiments, Section 4.2 details the preprocessing and postprocessing stages used in the forecasting process, Section 4.3 describes the procedure of parameters selection and Section 4.4 shows the performance metrics used in the experimental evaluation. Section 4.5 and Section 4.6 present the simulation results and some remarks are discussed, respectively.

### 4.1. Data Description

The energy consumption data used in this investigation were collected by a smart meter installed in a residential building located in New Taipei City (Taiwan) [64]. The residents are two adults and three children. The floor total area occupied is 350 m${}^{2}$.

The sampling used was 15 min for 30 days, from 22 June 2015 to 26 July 2015. Thus, four samples are recorded in one hour, with 96 points in one single day (24 h), and totaling 2880 points in 30 days. It is important to highlight that the original data set presented one missing sample, which was completed using the average of the neighbor points.

The data set was divide into three subsets, maintaining the temporal order: training (1824 samples or 19 days), validation (384 samples or four days), and test (672 samples or seven days). The AR model adjustment considered the first two subsets as one. Table 1 shows the statistical description of the whole series and the respective subsets.

### 4.2. Preprocessing and Postprocessing Stages

Energy consumption time series can be sampled weekly, daily, hourly, or minutely. As mentioned, the data used in this work was sampled every 15 min. This series presents a seasonal pattern every 96 points or an entire day. We performed a deseasonalization procedure that transforms the original series into approximately stationary, with zero mean and standard deviation close to one. This process changes the statistical behavior of the series, which may improve the output response of the forecasting models. In addition, the linear models from the Box and Jenkins methodology assume that the series is stationary [62]. The deseasonalization is given by Equation (10):

(10) $$z_{i,s}=\frac{x_{i,s}-{\hat{\mu }}_{s}}{{\hat{\sigma }}_{s}}$$

in which $z_{i,s}$ is the new standardized value of the *i* element of the original series $x_{i,s}$, ${\hat{\mu }}_{s}$ is the average of the elements of the series considering the seasonal pattern *s*, and ${\hat{\sigma }}_{s}$ is the standard deviation.

**Figure 2:**
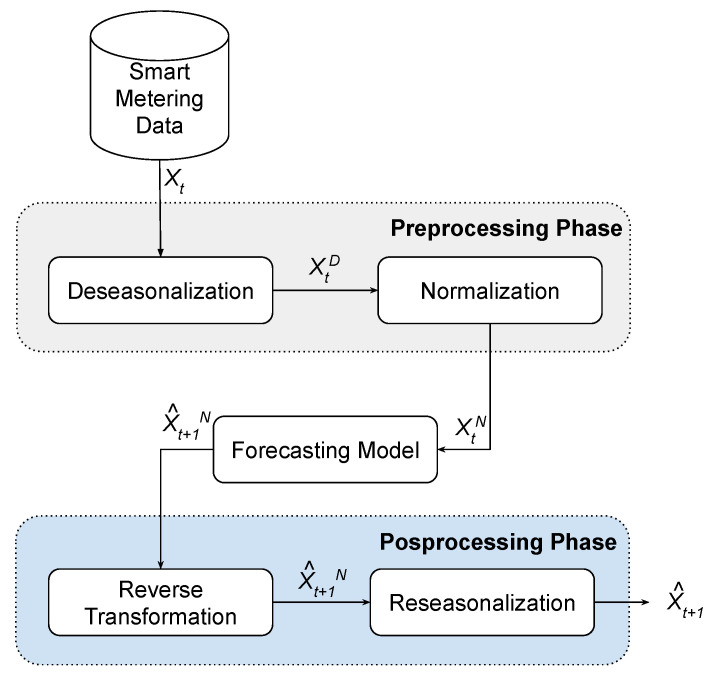
Stages of preprocessing and postprocessing employed in the modeling of the forecasting method.

All steps involved in the forecasting process of the consumption time series are summarized in Figure 2. The preprocessing stage is initiated after defining the time lags of the series, which are the inputs of the models. After that, deseasonalization is applied. Finally, a procedure to normalize the data into the interval [−1,+1] is performed. This normalization is mandatory for neural models that use hyperbolic tangent as activation function. After the preprocessing stage, the forecasting model generates the prediction based on the normalized inputs.

In the postprocessing stage, the normalization and deseasonalization are reversed, leading to time series data to the original domain. With forecasts in the original domain at hand, the comparison with the actual series is performed to evaluate the models’ errors.

### 4.3. Experimental Setup

In this investigation, the experimental evaluation was performed using as single methods AR, MLP, ELM, ESN, and RBF. The proposed ensemble method also was compared with three distinct combination approaches: the mean and the median of the single models’ outputs (non-trainable methods), and MLP [45,65].

As a baseline, we applied the seasonal autoregressive integrated moving average model (SARIMA) from the Box and Jenkins family [54], a classic linear model widely used in time series analysis. The parameters of the model were defined following the methodology proposed by Hyndman and Khandakar [66,67].

The parameters of the models addressed in this work were defined following some premises:

- The coefficients of the AR model were calculated using the Yule–Walker equations, a closed-form solution [54];
- All artificial neural networks used hyperbolic tangent as activation function of the hidden neurons [59];
- The number of neurons in the hidden layer was determined by previous empirical tests, considering a range of [3:500].
- All models were implemented in Matlab^®^.

Finally, The partial autocorrelation function (PACF) was applied to define the number of temporal lags used as inputs of the single models. Its application reveals that the first seven lags are significant, being adequate to solve the task. Therefore, all models employed a sliding window containing seven input lags in the forecasting of the electricity consumption dataset [54,68].

### 4.4. Error Metrics

For performing a comparative analysis among the forecasting models we adopted five error metrics: mean squared error (MSE), mean absolute error (MAE), mean absolute percentage error (MAPE), root mean squared error (RMSE), and index of agreement (IA), which are described in Equations (11) to (15), respectively [67]:

(11) $$MSE=\frac{1}{N}\sum {\left(x_{t}-{\hat{x}}_{t}\right)}^{2},$$

(12) $$MAE=\frac{1}{N}\sum \left|x_{t}-{\hat{x}}_{t}\right|,$$

(13) $$RMSE=\sqrt{\frac{1}{N}\sum {\left(x_{t}-{\hat{x}}_{t}\right)}^{2}},$$

(14) $$MAPE=\frac{100}{n}\sum \left|\frac{x_{t}-{\hat{x}}_{t}}{x_{t}}\right|,$$

(15) $$\mathrm{IA}=1-\frac{\sum_{t=1}^{N}{\left(x_{t}-{\hat{x}}_{t}\right)}^{2}}{\sum_{t=1}^{N}\left(\right|{\hat{x}}_{t}-\bar{x}\left|+\right|x_{t}-\bar{x}{\left|\right)}^{2}}.$$

In all equations, *N* is the number of samples, $x_{t}$ is the actual data, and ${\hat{x}}_{t}$ is the predicted sample in time *t*. The MSE is a quadratic error measure which penalizes higher errors, but is sensible to outliers. The RMSE is less sensible to outliers, since the root square of the MSE is calculated. Likewise, MAE offers an error metric closer do the scale of the data. Furthermore, MSE, RMSE, and MAE are scale dependent error metrics [67]. In contrast, MAPE and IA are not scale dependent. Note that all metrics must be minimized, except IA, which must be maximized in the range [0,1].

### 4.5. Results

Table 2 shows the values of the five error metrics (MSE, MAE, MAPE, RMSE, and IA) attained by the evaluated models for each day of the week. It is possible to observe that the approaches based on ensemble reached the best results in most of the cases (27 out of 35). The proposed ensemble ELM attained the best error values in 19 out of 35 comparisons. These results show the superiority of the proposal regarding statistical and ML models of the literature. The proposed ensemble obtained the best value in all weekdays in at least one performance metric. For instance, on Wednesday, Thursday, and Sunday, the ensemble ELM achieved the best values in majority of the performance measures. The single model ESN and the ensemble MLP reached the second-best result with the lowest error value in 5 out of 35 cases.

Considering the maximum value on all days of the week in Table 2, the ensemble ELM attained the best performance in terms of MAE, MAPE, and IA. Regarding the minimum value on all days of the week, the proposed ensemble achieved the best results in the MAE and IA metrics. The numbers show that the forecasts generated by the proposed ensemble presented stability on the different weekdays. These findings corroborate the hypothesis that supports the adoption of the ensemble in the forecasting task.

Table 3 shows the performance metrics values achieved by the models considering all days of the week. The results show that ensemble ELM attained the best values in all performance measures. The single ELM model obtained the second-best ranking in the MSE, MAE, RMSE, and IA metrics. The ensemble MLP attained the second-best MAPE value. The third-best value for the MSE, MAE, and RMSE were reached by the ensemble mean. The single models MLP and ESN achieved the third-best value for the MAPE and IA measures, respectively.

In order to verify if the proposed ensemble attained results statistically different from the other models, three hypothesis tests were used for this evaluation: Friedman test, Kruskal–Wallis test, and Wilcoxon test [69,70]. A significance level of 0.05 was employed in all hypothesis tests.

The statistical evaluation was performed from the MSE values obtained by the models in 30 independent executions, considering that some employed forecasting models, such as MLP, ELM, and ESN, have their parameters randomly initialized. In the literature, 30 samples are often considered sufficiently large and representative to perform the statistical analysis [71,72]. The *p*-values found were 2.65 $\times \;10^{-39}$ and 1.06 $\times \;10^{-43}$ for Friedman and Kruskal–Wallis tests, respectively. The Wilcoxon hypothesis test was employed to compare the results pairwise. In this case, the ensemble ELM (the best general model) and the ELM (the best single model) were compared with each forecasting model. Table 4 shows the *p*-values. In summary, considering the three tests addressed, we can assure that a change in the forecasting model led to distinct results since all p-values are smaller than 0.05.

### 4.6. Discussion

Many aspects can be discussed about the forecasting responses and errors presented in this evaluation. Table 5 was elaborated to present a ranking regarding the values of the metrics achieved by the predictors. The column *Mean* presents the average of the positions in the ranking considering all metrics, while column *Rank* presents a ranking ordering the predictors.

The most important result is that the proposed ensemble ELM achieved the best overall results considering all metrics. This result corroborates with assumptions that support the use of the ELM as the combination model. In addition, among the first four best predictors, we have three ensembles. However, as the results show, a change in the combiner may lead to poor performances, as presented by the MLP.

Comparing the single ML models, the ELM attained the best value in 4 out 5 performance metrics. Although the ELM is the “unorganized” version of the MLP neural network or a feedforward version of the ESN, the ELM model presented a superior accuracy among the single approaches. Furthermore, it is the second-best predictor, overcoming 3 of 4 ensembles employed in the experimental evaluation.

The autoregressive (AR) model attained the best result regarding the five performance metrics between the linear approaches. The AR also obtained a superior performance to the RBF model in all metrics and the MLP model in terms of MSE and RMSE. The results show that nonlinear ML models or ML-based ensembles are more appropriate for this kind of problem. The energy consumption time series can have nonlinear patterns [7] that are not properly modeled by linear techniques, such as AR or SARIMA. However, ML models’ adoption can also lead to underperforming results due to problems regarding overfitting, underfitting, or misspecification [59]. These issues can be related to the poor performance of the RBF model or the result of the combination using the MLP model that was not able to overcome the nontrainable ensembles (ensemble mean and ensemble median). It is also important to mention that the computational cost to adjust the ELM and ESN is smaller than the fully trained models, such as MLP and RBF.

**Figure 3:**
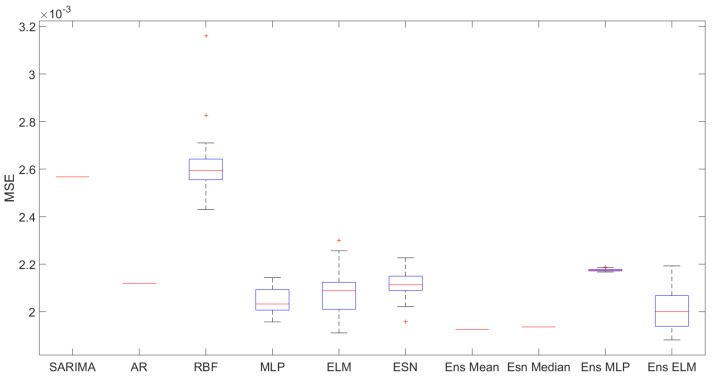
Boxplot graphic.

Figure 3 presents the boxplot of 30 simulations of each predictor. As expected, the SARIMA, AR, ensemble mean, and ensemble median do not present dispersion, since they present closed form solutions. It is interesting to note that the MLP showed a small dispersion, followed by the ESN. Regarding the median of the values, it is important to mention that the 3 best ensembles presented the best results, followed by the MLP.

**Figure 4:**
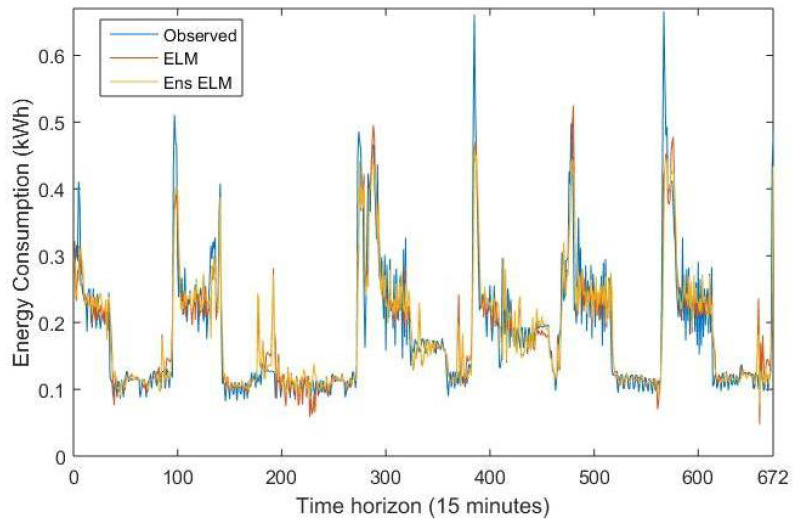
Energy consumption forecasting obtained by the ELM and ensemble ELM.

Finally, Figure 4 depicts the real energy consumption time series, and the forecasting provided by the ensemble ELM (the best overall predictor) and the ELM, the best single model.

## 5. Conclusions

Energy consumption time series may present present linear and non-linear patterns, which hinders models to achieve accurate predictions. In this sense, the use of ensembles stood out in the current literature due to their high capability to increase the prediction power of stand-alone forecasting models. Based on that, we propose in this work neural-based ensembles for energy consumption forecasting.

More specifically, we use in this investigation as predictors the linear AR, and neural networks architectures: MLP, ELM, ESN, and RBF. As a combiner, we employ the non-trainable ensembles based on mean and median, and the MLP and ELM. As a baseline we address the SARIMA model.

The experimental evaluation was conduced by using a series from a residential building containing a installed smart grid network. Before the simulations, we applied a deseasonalization procedure in order to make the series stationary. The computational results showed that the ELM-based ensemble outperformed the other proposals in terms of five distinct error metrics. In addition, the single ELM stood out in comparison to the other single approaches. This is an important observation, since the ELM is a neural network with a simple training process, which confers a fast adjustment of the free parameters of the architecture.

As possible future directions, variable selection techniques can be applied to define the best lags as inputs of the forecasting models, and error correction hybrid models can be used to produce more accurate models. Finally, the propositions of this work should be tested in other databases related to energy consumption.

## Figures and Tables

**Table 1:** Mean and standard deviation of the sets.

Set	Number of Samples	Mean (kWh)	Standard Deviation
Whole Series	2880	0.20077	0.10115
Training	1824	0.20794	0.10238
Validation	384	0.19789	0.10065
Test	672	0.18296	0.09579

**Table 2:** The performance results in terms of the MSE, MAE, MAPE, RMSE, and IA metrics of the proposed Ensemble and literature models for each day of the week. The best values are highlighted in bold.

	Model	Measure	Monday	Tuesday	Wednesday	Thursday	Friday	Saturday	Sunday	Max	Min
Single	SARIMA	MSE ($\times 10^{-3}$ kWh)	2.8720	2.7614	3.3822	2.9381	1.7513	2.0152	2.2561	3.3822	1.7513
		MAE (kWh)	0.0325	0.0332	0.0359	0.0353	0.0217	0.0288	0.0246	0.0359	0.0217
		MAPE (%)	15.8363	14.2251	17.1321	19.2733	13.2665	16.0358	14.9922	19.2733	13.2665
		RMSE (kWh)	0.0535	0.0525	0.0581	0.0542	0.0418	0.0448	0.0474	0.0581	0.0418
		IA	0.8400	0.8991	0.9272	0.8162	0.8970	0.9296	0.9419	0.9419	0.8162
	AR	MSE ($\times 10^{-3}$ kWh)	1.1708	1.8802	1.9812	1.8478	2.2187	3.4516	2.2879	3.4516	1.1708
		MAE (kWh)	0.0225	0.0297	0.0234	0.0300	0.0281	0.0355	0.0337	0.0355	0.0225
		MAPE (%)	14.3075	17.0181	14.9965	16.1628	**11.8905**	15.8668	19.7286	19.7286	**11.8905**
		RMSE (kWh)	0.0342	0.0434	0.0445	0.0430	0.0471	0.0588	0.0478	0.0588	0.0342
		IA	0.9355	0.9304	0.9568	0.9027	0.9202	0.9285	0.8827	0.9568	0.8827
	MLP	MSE ($\times 10^{-3}$ kWh)	1.1413	1.7036	1.8264	1.7168	2.1059	3.2103	1.9979	3.2103	1.1413
		MAE (kWh)	0.0217	0.0282	0.0216	0.0286	0.0289	0.0332	0.0304	0.0332	0.0216
		MAPE (%)	13.5270	15.7165	12.6500	15.4307	12.3867	14.9471	17.9853	17.9853	12.3867
		RMSE (kWh)	0.0338	0.0413	0.0427	0.0414	0.0459	0.0567	0.0447	0.0567	0.0338
		IA	**0.9367**	0.9375	0.9583	0.9100	0.9260	0.9325	0.9000	0.9583	0.9000
	ELM	MSE ($\times 10^{-3}$ kWh)	1.1526	1.6701	1.7890	1.7343	2.0591	3.1423	1.8294	3.1423	1.1526
		MAE (kWh)	**0.0209**	0.0271	0.0222	0.0285	0.0292	0.0329	0.0287	0.0329	0.0209
		MAPE (%)	12.7378	15.2818	13.9267	15.4566	12.6752	14.6467	17.0593	17.0593	12.6752
		RMSE (kWh)	0.0340	0.0409	0.0423	0.0416	0.0454	0.0561	0.0428	0.0561	0.0340
		IA	0.9356	0.9383	0.9594	0.9091	0.9284	0.9322	0.9079	0.9594	0.9079
	ESN	MSE ($\times 10^{-3}$ kWh)	1.1806	**1.5424**	1.7851	1.7948	1.9928	3.3204	2.0896	3.3204	1.1806
		MAE (kWh)	0.0213	0.0269	0.0220	0.0293	**0.0279**	0.0336	0.0305	0.0336	0.0213
		MAPE (%)	12.9235	15.4666	13.9666	15.9643	11.9069	14.9907	18.2586	18.2586	11.9069
		RMSE (kWh)	0.0344	**0.0393**	0.0423	0.0424	0.0446	0.0576	0.0457	0.0576	0.0344
		IA	0.9345	**0.9460**	0.9605	0.9044	**0.9320**	0.9320	0.8964	0.9605	0.8964
	RBF	MSE ($\times 10^{-3}$ kWh)	1.7832	1.7691	3.0669	2.1245	2.2380	3.4564	2.5668	3.4564	1.7691
		MAE (kWh)	0.0261	0.0287	0.0326	0.0313	0.0313	0.0368	0.0322	0.0368	0.0261
		MAPE (%)	15.2994	15.4352	22.0301	17.4932	13.7047	17.3610	20.1081	22.0301	13.7047
		RMSE (kWh)	0.0422	0.0421	0.0554	0.0461	0.0473	0.0588	0.0507	0.0588	0.0421
		IA	0.8957	0.9364	0.9243	0.8828	0.9245	0.9242	0.8633	0.9364	0.8633
Ensemble	Ensemble Mean	MSE ($\times 10^{-3}$ kWh)	1.1632	1.6345	1.8150	1.7466	2.0379	3.1300	1.9460	3.1300	1.1632
		MAE (kWh)	0.0216	0.0277	0.0220	0.0289	0.0282	0.0325	0.0300	0.0325	0.0216
		MAPE (%)	13.0506	15.4767	13.5962	15.7273	12.1594	14.2690	17.7973	17.7973	12.1594
		RMSE (kWh)	0.0341	0.0404	0.0426	0.0418	0.0451	0.0559	0.0441	0.0559	0.0341
		IA	0.9342	0.9404	0.9583	0.9061	0.9289	**0.9336**	0.8992	0.9583	0.8992
	Ensemble Median	MSE ($\times 10^{-3}$ kWh)	**1.1378**	1.6290	1.8093	1.7949	2.0108	3.1865	1.9856	3.1865	**1.1378**
		MAE (kWh)	0.0215	0.0279	0.0214	0.0294	0.0281	0.0332	0.0303	0.0332	0.0214
		MAPE (%)	13.2260	15.7893	13.1504	15.9486	12.1569	14.8732	17.9705	17.9705	12.1569
		RMSE (kWh)	**0.0337**	0.0404	0.0425	0.0424	0.0448	0.0564	0.0446	0.0564	**0.0337**
		IA	0.9365	0.9409	0.9593	0.9046	0.9291	0.9329	0.8992	0.9593	0.8992
	Ensemble MLP	MSE ($\times 10^{-3}$ kWh)	1.1588	1.5856	1.7507	**1.7038**	1.9816	**3.0892**	1.7540	**3.0892**	1.1588
		MAE (kWh)	0.0210	0.0266	0.0219	**0.0278**	0.0292	0.0319	0.0275	0.0319	0.0210
		MAPE (%)	12.7435	**14.9182**	13.5774	15.2293	12.6736	14.1799	16.2368	16.2368	12.6736
		RMSE (kWh)	0.0340	0.0398	0.0418	0.0413	0.0445	**0.0556**	0.0419	**0.0556**	0.0340
		IA	0.9357	0.9428	0.9592	0.9095	0.9300	0.9328	0.9117	0.9592	0.9095
	Ensemble ELM	MSE ($\times 10^{-3}$ kWh)	1.2162	1.7898	**1.5592**	1.7071	**1.9356**	3.4034	**1.5598**	3.4034	1.2162
		MAE (kWh)	0.0217	**0.0261**	**0.0203**	0.0278	0.0303	**0.0296**	**0.0243**	**0.0303**	**0.0203**
		MAPE (%)	**12.5109**	15.0994	**12.1248**	**14.8702**	13.2287	**13.0881**	**13.8786**	**15.0994**	12.1248
		RMSE (kWh)	0.0349	0.0423	**0.0395**	**0.0413**	**0.0440**	0.0583	**0.0395**	0.0583	0.0349
		IA	0.9321	0.9382	**0.9624**	**0.9124**	0.9300	0.9249	**0.9263**	**0.9624**	**0.9124**

**Table 3:** MSE, MAE, MAPE, RMSE, and IA values for the evaluated models. The number of neurons used by each neural network is shown in the NN column. The performance corresponds to the whole test set of the energy consumption series. The best value for each metric is highlighted in bold.

	Model	NN	MSE ($\times 10^{-3}$ kWh)	MAE (kWh)	MAPE (%)	RMSE (kWh)	IA
Single	SARIMA	-	2.5675	0.0303	15.4004	0.0506	0.9129
	AR	-	2.1195	0.0290	15.7090	0.0460	0.9318
	MLP	200	1.9574	0.0275	14.5376	0.0442	0.9391
	ELM	120	1.9110	0.0271	14.5393	0.0437	0.9405
	ESN	40	1.9579	0.0274	14.7819	0.0442	0.9402
	RBF	60	2.4292	0.0310	17.1017	0.0493	0.9226
Ensemble	Ensemble Mean	-	1.9247	0.0273	14.5826	0.0439	0.9373
	Ensemble Median	-	1.9363	0.0274	14.7307	0.0440	0.9375
	Ensemble MLP	40	2.1671	0.0284	14.2228	0.0466	0.9358
	Ensemble ELM	60	**1.8817**	**0.0257**	**13.5424**	**0.0434**	**0.9410**

**Table 4:** *p*-values of the Wilcoxon statistical test comparing the Ensemble ELM and ELM with the other forecasting models.

Models	*p*-Value (Ensemble ELM)	*p*-Value (ELM)
Ensemble ELM	—	0.0013
ELM	0.0013	—
SARIMA	1.21$\;\times \;10^{-12}$	1.21$\;\times \;10^{-12}$
AR	7.47$\;\times \;10^{-10}$	0.0045
MLP	0.0241	0.0323
ESN	1.72$\;\times \;10^{-6}$	0.0478
RBF	3.01$\;\times \;10^{-11}$	3.01$\;\times \;10^{-11}$
Ensemble Mean	1.91$\;\times \;10^{-7}$	3.35$\;\times \;10^{-11}$
Ensemble Median	2.05$\;\times \;10^{-5}$	3.35$\;\times \;10^{-11}$
Ensemble MLP	8.48$\;\times \;10^{-9}$	1.35$\;\times \;10^{-7}$

**Table 5:** Ranking of the models for each performance metric in the energy consumption forecasting.

	Model	MSE (kWh)	MAE (kWh)	MAPE (%)	RMSE (kWh)	IA	Mean	Rank
Single	SARIMA	10	9	8	10	10	9.4	9
	AR	7	8	9	7	8	7.6	8
	MLP	5	6	3	5	4	4.6	5
	ELM	2	2	4	2	2	2.4	2
	ESN	6	4	7	6	3	5.2	6
	RBF	9	10	10	9	9	9.4	9
Ensemble	Ensemble Mean	3	3	5	3	6	4	3
	Ensemble Median	4	5	6	4	5	4.8	4
	Ensemble MLP	8	7	2	8	7	6.4	7
	Ensemble ELM	1	1	1	1	1	1	1

## Data Availability

The authors would like to thank the database provided by Jui-Sheng Chou.
